# Lessons from a misdiagnosed maternal pulmonary embolism: A case report and literature review

**DOI:** 10.1097/MD.0000000000046918

**Published:** 2026-01-02

**Authors:** Pei Chi, Rongxing Shi, Ting Wu, Meng-Jie Li, Hanlong Guo, Qu-Cheng Huang, Wen-Juan Liao

**Affiliations:** a Department of Obstetrics, Renmin Hospital of Qingxian, Cangzhou, China; b Department of Thoracic Surgery, Nanjing Drum Tower Hospital Clinical College of Nanjing Medical University, Nanjing, China; c Department of Thyroid and Breast Surgery, General Hospital of Central Theater Command, Wuhan, China; d Department of Pulmonary and Critical Care Medicine, Renmin Hospital of Qingxian, Cangzhou, China; e Department of the First Clinical Medical College, Gannan Medical University, Ganzhou, China; f Department of Child, The First Affiliated Hospital of Gannan Medical University, Ganzhou, China.

**Keywords:** case report, glucocorticoid, maternal, misdiagnosis, pulmonary embolism

## Abstract

**Rationale:**

Pulmonary embolism (PE) is a leading cause of maternal mortality in developed countries. However, because its clinical symptoms overlap significantly with normal physiological changes during pregnancy, it is extremely prone to misdiagnosis.

**Patient concerns:**

A pregnant woman in the early stage of pregnancy was diagnosed with pneumonia due to fever and cough. After ineffective treatment with penicillin, her symptoms were relieved after she was switched to cephalosporin antibiotics combined with methylprednisolone. However, the symptoms recurred after discontinuation of the medication.

**Diagnoses:**

Further chest contrast-enhanced computed tomography confirmed PE.

**Interventions:**

Through active anticoagulation treatment, the patient’s symptoms completely recovered, and her abnormal imaging manifestations also significantly decreased.

**Outcomes:**

Eventually, the woman gave birth to a healthy male baby.

**Lessons:**

The process of misdiagnosis in this case was complicated and worthy of further investigation. These findings suggest that antibiotics combined with methylprednisolone can alleviate the symptoms of early pregnancy PE, but it should not be used as the main treatment method.

## 1. Introduction

Pulmonary embolism (PE) in the early stages of pregnancy is often misdiagnosed because of its atypical symptoms, which can overlap significantly with normal physiological changes during pregnancy. This diagnostic challenge is compounded by the fact that symptoms such as tachycardia, tachypnea, and shortness of breath are common in both normal pregnancies and PE, making it difficult to distinguish between the two without further diagnostic testing.^[1]^ Physiological changes during pregnancy, such as increased blood volume and cardiac output, can mask the clinical signs of PE, leading to delays in diagnosis and treatment.^[2,3]^ This delay is particularly concerning given that PE is a leading cause of maternal mortality in developed countries.^[4]^ The diagnostic process for PE in pregnant women is further complicated by the limitations of traditional diagnostic tools.^[5–7]^

## 2. Case report

A 28-year-old married housewife presented to the hospital with amenorrhea for more than 2 months and fever with coughing for 1 week. She had no underlying disease. After amenorrhea, her blood human chorionic gonadotropin test and ultrasound examination confirmed early pregnancy (a single viable fetus). During the early stage of pregnancy, she had no obvious early pregnancy reactions, no abdominal pain or abdominal distension, and no vaginal bleeding or abnormal discharge. In the past week, she developed intermittent fever without obvious cause, with the highest temperature reaching 38.0°C. She had no nausea, vomiting, or night sweats. She had intermittent coughing, which was a dry, irritating cough with no obvious sputum. She denied contact with sources of epidemic infection and had no travel history.

**Figure 1.**
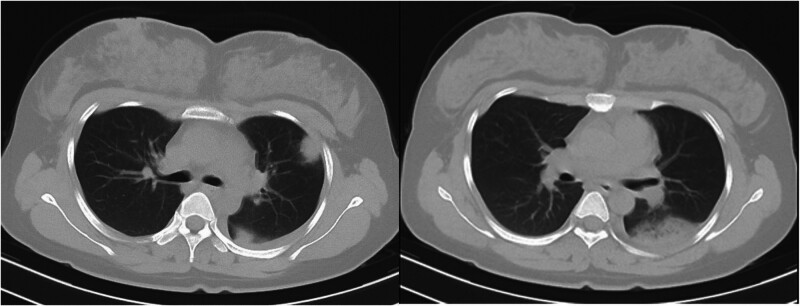
Sixteen-slice computerized tomography images of the chest revealed localized patchy shadows in the left lung.

Physical examination revealed that she was conscious, with a slightly congested pharynx, coarse breath sounds in both lungs, no dry or wet rales, no bulges in the precordium, no palpable tremors, a heart rate of 116 beats per minute, a regular rhythm, and no murmurs in the auscultation areas of the heart valves. Her abdomen was flat, and there was no edema in either lower extremity. Laboratory tests revealed that her white blood cell count increased to 15.00 × 10^9^/L (normal range: 3.5–9.5 × 10^9^/L), and her neutrophil count was 72.9%. The C-reactive protein level was 165.12 mg/L, the erythrocyte sedimentation rate was 75.00 mm/h, and the procalcitonin level was normal. Liver and kidney functions and blood glucose levels were normal. She underwent a chest computed tomography (CT) scan with abdominal radiation protection, which revealed bilateral pneumonia and left pleural effusion with multiple peripheral patchy high-density shadows in the left lung and a few signs of bronchial air (Fig. 1). The initial diagnosis was respiratory tract infection. She was treated with 4.8 million units of penicillin intravenously once every 8 hours.

After 3 days of treatment, her fever symptoms worsened, with her temperature rising to 40.0°C several times, and her cough also worsened, accompanied by hemoptysis. After discussion by the multidisciplinary team, the patient believed that her CT imaging manifestations were acute organizing pneumonia. The antibiotic was changed to 2.0 g of ceftriaxone intravenously once a day, and 20 mg of methylprednisolone was added intravenously once a day. After 1 week of treatment, her symptoms improved, she no longer had fever, and her cough symptoms also significantly decreased, with no sputum.

**Figure 2.**
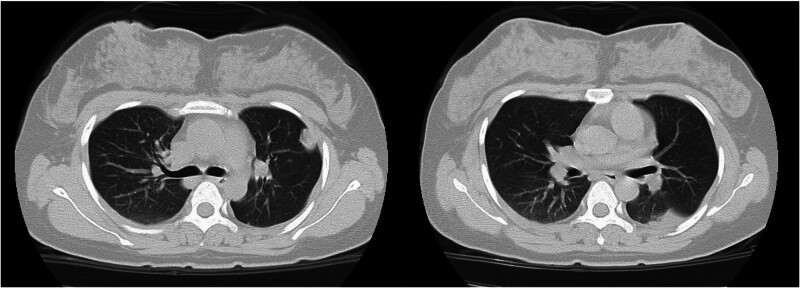
Ten days later, 16-slice computed tomography reexamination images of the chest revealed that the shadow in the patient’s lungs decreased and became lighter in the left lung after treatment with a combination of antibiotics and methylprednisolone.

**Figure 3.**
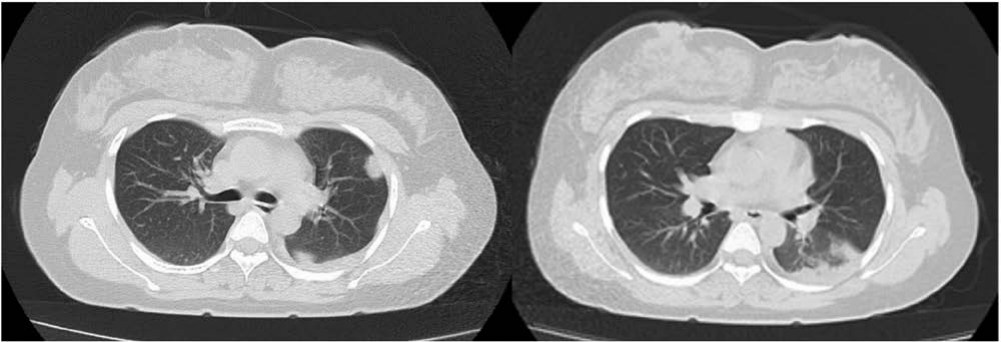
Three weeks later, 16-slice computerized tomography reexamination images of the chest revealed reexpansion of the shadows on the CT images after antibiotic and methylprednisolone withdrawal.

**Figure 4.**
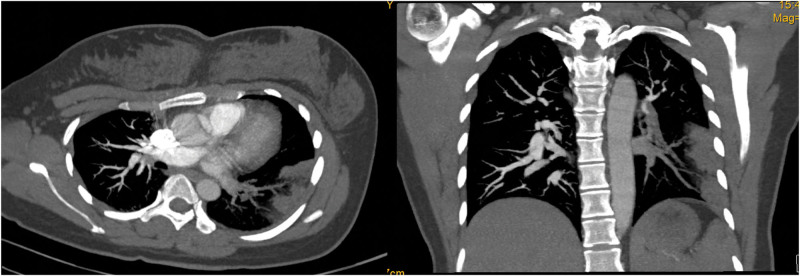
Sixteen-slice contrast-enhanced computerized tomography images of the chest revealed pulmonary embolism.

The dose of methylprednisolone was gradually reduced and stopped after 1 week. A reexamination of the chest CT image revealed that the range of patchy consolidation in the left lung was significantly reduced, and most of the left pleural effusion was absorbed (Fig. 2). However, 3 days after stopping methylprednisolone, she developed fever again, with a temperature of 38.5°C and a heart rate of 119 beats per minute. A reexamination of the chest CT revealed that the range of consolidation in the left lung had expanded compared with before, and there was a small amount of pleural effusion in the left pleural cavity (Fig. 3). Owing to the poor treatment effect of pneumonia, her lung consolidation decreased after hormone use and then increased again, and her heart rate remained fast. She underwent a chest contrast-enhanced CT scan, which revealed bilateral pulmonary artery embolism, poor perfusion in most of the left lower lobe pulmonary artery branches, and partial lung infarction in the left lung (Fig. 4). She underwent blood biochemical tests, which revealed no obvious abnormal values. She also underwent blood d-dimer testing, which increased to 2.97 mg/L (normal value < 0.3 mg/L). She underwent echocardiography and lower extremity vascular ultrasound, which revealed no obvious abnormalities.

**Figure 5.**
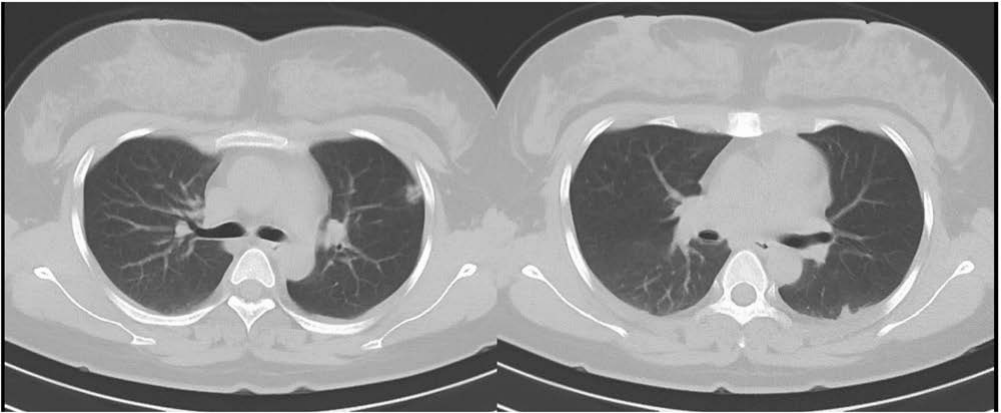
During the follow-up period, 16-slice computerized tomography reexamination images of the chest revealed significant absorption of the lung infarction lesion.

Subsequently, she received 20 mg rivaroxaban orally once a day for anticoagulation treatment. One week later, her symptoms gradually disappeared. Two weeks later, a reexamination of the chest CT image revealed significant absorption of the lung infarction lesion (Fig. 5). She was subsequently followed up for 5 months and did not experience the above symptoms again. Finally, she successfully gave birth to a healthy baby boy.

## 3. Discussion

PE is a critical medical condition characterized by the obstruction of pulmonary arteries due to blood clots. The incidence of PE in maternal populations is a significant concern because of its potential to cause severe morbidity and mortality. The chance of experiencing PE is 6 times greater for pregnant women than for those who are not pregnant.^[8]^ Studies suggest that the incidence of PE is greater in the puerperium period than in the deep vein thrombosis period.^[9]^

PE during pregnancy is a critical condition that poses significant diagnostic challenges because its symptoms overlap with normal physiological changes during pregnancy. The diagnosis of PE in pregnant women is further complicated by the limitations of traditional diagnostic tools and the scarcity of high-quality research specifically targeting this population. Common symptoms of PE, such as tachycardia, shortness of breath and breathing difficulties, are also common in normal pregnancy and preeclampsia. This makes it difficult to distinguish among them without further diagnostic tests. Physiological changes during pregnancy, such as increased blood volume and increased cardiac output, may mask the clinical symptoms of PE, leading to delays in diagnosis and treatment. The diagnosis of PE during pregnancy is fraught with challenges, especially because of the physiological changes associated with the potential risks associated with diagnostic imaging. One of the central issues in diagnosing PE during pregnancy is the overreliance on computed tomographic pulmonary angiography, which, while accurate, carries risks of radiation exposure to both the mother and fetus.^[10,11]^ Misdiagnosis or overdiagnosis of PE can lead to significant health implications for both the mother and fetus.

In this case, the misdiagnosis of PE during pregnancy needs to be analyzed from the aspects of symptoms, diagnosis and treatment. In terms of symptoms, the patient’s chief complaint was fever accompanied by an irritating cough, which is not a characteristic clinical symptom of PE (chest pain, hemoptysis, and dyspnea). In terms of diagnosis, CT imaging and laboratory tests all indicated a tendency toward pulmonary infectious lesions. Although the fever and cough symptoms did not resolve during the penicillin treatment stage, they were relieved after treatment with cephalosporins and methylprednisolone. The CT imaging results also improved. However, the symptoms worsened rapidly after the medication was stopped. Through careful observation of the chest CT images, it was found that the manifestations of previously misdiagnosed lung pneumonia were worthy of deep consideration. First, the lung shadow in this case was wedge-shaped consolidation, with the base facing the pleura and the tip pointing toward the hilum, which is a wedge-shaped shadow similar to Hampton hump, rather than the typical imaging features of pneumonia (irregular patchy shadows, consolidation distributed in lobes or segments, with blurred boundaries, and no specific geometric shape). Second, the lung shadow in this case contained a stiff air bronchogram, which is not the smooth and natural air bronchogram seen in pneumonia cases. In terms of treatment, the combination of antibiotics and methylprednisolone in treating this misdiagnosed case of PE achieved symptomatic and radiological relief, which cannot be acknowledged but can be explained. Methylprednisolone can inhibit the expression of vascular endothelial growth factor, thereby reduce vascular permeability and stabilize the tight junctions of the capillary endothelium, leading to a reduction in red blood cell exudation.^[12–14]^ Additionally, methylprednisolone can inhibit inflammatory exudation, resulting in a reduction in the range of pulmonary consolidation on CT images, but it cannot reverse the necrotic core of pulmonary infarction.^[15,16]^ This further explains the reexpansion of the lesion on CT images after drug withdrawal. Moreover, methylprednisolone can alleviate hemoptysis symptoms. These factors led to the delayed diagnosis of PE in this case.

The diagnostic process for PE in pregnant women is further complicated by the limitations of traditional diagnostic tools. The d-dimer test, which is commonly used to rule out PE in the general population, loses its diagnostic accuracy during pregnancy because of the physiological increase in d-dimer levels.^[5]^ A systematic review and meta-analysis examining the diagnostic accuracy of d-dimer in peripartum patients underscores these challenges, which found that while d-dimer tests in the peripartum period exhibit high sensitivity for detecting venous thromboembolism, their specificity is considerably lower, with an area under the receiver operating characteristic curve of only 0.76.^[17]^ This suggests that while the test is effective at identifying true positive cases, it also results in a high number of false positives, limiting its clinical applicability without additional diagnostic tools or decision rules.^[17]^ In addition to these challenges, research on the use of d-dimer tests in other contexts, such as adnexal torsion in pregnant women, further illustrates the variability in diagnostic accuracy. A study evaluating serum d-dimer levels in pregnant women with adnexal torsion found that while elevated d-dimer levels were associated with adnexal torsion, the test’s sensitivity and specificity varied significantly depending on the clinical context.^[18]^

Additionally, imaging modalities such as computed tomographic pulmonary angiography and ventilation–perfusion (V/Q) scans, while effective, involve radiation exposure, which poses potential risks to the fetus.^[3,6]^ Despite these challenges, maintaining a high degree of suspicion for PE in pregnant patients and utilizing appropriate diagnostic strategies to ensure timely and accurate diagnosis are crucial.^[7]^

International guidelines for diagnosing PE in pregnancy vary significantly, reflecting the scarcity of high-quality research in this area. A comprehensive review of these guidelines reveals conflicting recommendations, particularly regarding the use of clinical prediction tools, risk stratification, and imaging modalities.^[4]^ This inconsistency highlights the need for further research to establish evidence-based protocols that can be universally applied. Therefore, given the present circumstances, the integration of clinical symptoms, d-dimer testing, lower extremity vascular ultrasound examination, along with consideration of factors such as obesity, constitutes a fundamental approach for the clinical diagnosis of PE, enabling clinicians to either suspect or exclude this condition.

## 4. Limitations

This was a case report study, which was not highlight the long follow-up period, and diagnosis have limitations.

## 5. Conclusion

In conclusion, this case illustrates that the diagnostic process for PE in pregnant women is further complicated by the limitations of traditional diagnostic tools. These findings suggest that antibiotics combined with methylprednisolone can alleviate the symptoms of early pregnancy PE, but it should not be used as the main treatment method.

## Author contributions

**Conceptualization:** Rongxing Shi.

**Data curation:** Pei Chi, Meng-Jie Li, Wen-Juan Liao.

**Formal analysis:** Pei Chi, Ting Wu.

**Funding acquisition:** Rongxing Shi, Wen-Juan Liao.

**Investigation:** Ting Wu, Qu-Cheng Huang, Wen-Juan Liao.

**Methodology:** Hanlong Guo, Wen-Juan Liao.

**Project administration:** Ting Wu, Wen-Juan Liao.

**Resources:** Qu-Cheng Huang.

**Supervision:** Rongxing Shi, Ting Wu.

**Validation:** Rongxing Shi.

**Visualization:** Qu-Cheng Huang.

**Writing – review & editing:** Rongxing Shi, Meng-Jie Li, Hanlong Guo, Wen-Juan Liao.

**Writing – original draft:** Pei Chi, Ting Wu, Qu-Cheng Huang, Wen-Juan Liao.
